# The association between low skeletal muscle mass and delirium: results from the nationwide multi-centre Italian Delirium Day 2017

**DOI:** 10.1007/s40520-021-01950-8

**Published:** 2021-08-20

**Authors:** Alberto Zucchelli, F. Manzoni, A. Morandi, S. Di Santo, E. Rossi, M. G. Valsecchi, M. Inzitari, A. Cherubini, M. Bo, E. Mossello, A. Marengoni, G. Bellelli, the Italo-Hispanic Study Group of Delirium, A. Tarasconi, M. Sella, S. Auriemma, G. Paternò, G. Faggian, C. Lucarelli, N. De Grazia, C. Alberto, A. Margola, L. Porcella, I. Nardiello, E. Chimenti, M. Zeni, A. Giani, S. Famularo, E. Romairone, C. Minaglia, C. Ceccotti, G. Guerra, G. Mantovani, F. Monacelli, C. Minaglia, T. Candiani, A. Ballestrero, C. Minaglia, F. Santolini, C. Minaglia, M. Rosso, V. Bono, S. Sibilla, P. Dal Santo, M. Ceci, P. Barone, T. Schirinzi, A. Formenti, G. Nastasi, G. Isaia, D. Gonella, A. Battuello, S. Casson, D. Calvani, F. Boni, A. Ciaccio, R. Rosa, G. Sanna, S. Manfredini, L. Cortese, M. Rizzo, R. Prestano, A. Greco, M. Lauriola, G. Gelosa, V. Piras, M. Arena, D. Cosenza, A. Bellomo, M. LaMontagna, L. Gabbani, L. Lambertucci, S. Perego, G. Parati, G. Basile, V. Gallina, G. Pilone, C. Giudice, F. De, L. Pietrogrande, B. De, M. Mosca, I. Corazzin, P. Rossi, V. Nunziata, F. D‘Amico, A. Grippa, S. Giardini, R. Barucci, A. Cossu, L. Fiorin, M. Arena, M. Distefano, M. Lunardelli, M. Brunori, I. Ruffini, E. Abraham, A. Varutti, E. Fabbro, A. Catalano, G. Martino, D. Leotta, A. Marchet, G. Dell‘Aquila, A. Scrimieri, M. Davoli, M. Casella, A. Cartei, G. Polidori, G. Basile, D. Brischetto, S. Motta, R. Saponara, P. Perrone, G. Russo, D. Del, C. Car, T. Pirina, S. Franzoni, A. Cotroneo, F. Ghiggia, G. Volpi, C. Menichetti, M. Bo, A. Panico, P. Calogero, G. Corvalli, M. Mauri, E. Lupia, R. Manfredini, F. Fabbian, A. March, M. Pedrotti, M. Veronesi, E. Strocchi, A. Bianchetti, A. Crucitti, V. Di Francesco, G. Fontana, L. Bonanni, F. Barbone, C. Serrati, G. Ballardini, M. Simoncelli, G. Ceschia, C. Scarpa, R. Brugiolo, S. Fusco, T. Ciarambino, C. Biagini, E. Tonon, M. Porta, D. Venuti, M. DelSette, M. Poeta, G. Barbagallo, G. Trovato, A. Delitala, P. Arosio, F. Reggiani, G. Zuliani, B. Ortolani, E. Mussio, A. Girardi, A. Coin, G. Ruotolo, A. Castagna, M. Masina, R. Cimino, A. Pinciaroli, G. Tripodi, U. Cannistrà, F. Cassadonte, M. Vatrano, F. Cassandonte, L. Scaglione, P. Fogliacco, C. Muzzuilini, F. Romano, A. Padovani, L. Rozzini, A. Cagnin, F. Fragiacomo, G. Desideri, E. Liberatore, A. Bruni, G. Orsitto, M. Franco, L. Bonfrate, M. Bonetto, N. Pizio, G. Magnani, G. Cecchetti, A. Longo, V. Bubba, L. Marinan, M. Cotelli, M. Turla, M. Brunori, M. Sessa, L. Abruzzi, G. Castoldi, D. LoVetere, C. Musacchio, M. Novello, A. Cavarape, A. Bini, A. Leonardi, F. Seneci, W. Grimaldi, F. Fimognari, V. Bambara, A. Saitta, F. Corica, M. Braga, E. Ettorre, C. Camellini, A. Marengoni, A. Bruni, A. Crescenzo, G. Noro, R. Turco, M. Ponzetto, L. Giuseppe, B. Mazzei, G. Maiuri, D. Costaggiu, R. Damato, E. Fabbro, G. Patrizia, L. Santuari, M. Gallucci, C. Minaglia, M. Paragona, P. Bini, D. Modica, C. Abati, M. Clerici, I. Barbera, F. NigroImperiale, A. Manni, C. Votino, C. Castiglioni, M. Di, M. Degl‘Innocenti, G. Moscatelli, S. Guerini, C. Casini, D. Dini, S. DeNotariis, F. Bonometti, C. Paolillo, A. Riccardi, A. Tiozzo, A. SamySalamaFahmy, A. Riccardi, C. Paolillo, M. DiBari, S. Vanni, A. Scarpa, D. Zara, P. Ranieri, P. Calogero, G. Corvalli, D. Pezzoni, S. Gentile, A. Morandi, C. Platto, V. D‘Ambrosio, B. Faraci, C. Ivaldi, P. Milia, F. DeSalvo, C. Solaro, M. Strazzacappa, M. Bo, A. Panico, M. Cazzadori, S. Confente, M. Bonetto, G. Magnani, G. Cecchetti, V. Guerini, B. Bernardini, C. Corsini, S. Boffelli, A. Filippi, K. Delpin, E. Bertoletti, M. Vannucci, F. Tesi, P. Crippa, A. Malighetti, C. Caltagirone, S. DiSant, D. Bettini, F. Maltese, M. Formilan, G. Abruzzese, C. Minaglia, D. Cosimo, M. Azzini, M. Cazzadori, M. Colombo, G. Procino, S. Fascendini, F. Barocco, P. Del, F. D‘Amico, A. Grippa, A. Mazzone, E. Riva, D. Dell‘Acqua, M. Cottino, G. Vezzadini, S. Avanzi, S. Orini, F. Sgrilli, A. Mello, L. Lombardi, E. Muti, B. Dijk, S. Fenu, C. Pes, P. Gareri, A. Castagna, M. Passamonte, F. De, R. Rigo, L. Locusta, L. Caser, G. Rosso, S. Cesarini, R. Cozzi, C. Santini, P. Carbone, I. Cazzaniga, R. Lovati, A. Cantoni, P. Ranzani, D. Barra, G. Pompilio, S. Dimori, S. Cernesi, C. Riccò, F. Piazzolla, E. Capittini, C. Rota, F. Gottardi, L. Merla, A. Barelli, A. Millul, G. De, G. Morrone, M. Bigolari, C. Minaglia, M. Macchi, F. Zambon, F. D‘Amico, F. D‘Amico, C. Pizzorni, G. DiCasaleto, G. Menculini, M. Marcacci, G. Catanese, D. Sprini, T. DiCasalet, M. Bocci, S. Borga, P. Caironi, C. Cat, E. Cingolani, L. Avalli, G. Greco, G. Citerio, L. Gandini, G. Cornara, R. Lerda, L. Brazzi, F. Simeone, M. Caciorgna, D. Alampi, S. Francesconi, E. Beck, B. Antonini, K. Vettoretto, M. Meggiolaro, E. Garofalo, A. Bruni, S. Notaro, R. Varutti, F. Bassi, G. Mistraletti, A. Marino, R. Rona, E. Rondelli, I. Riva, A. Scapigliati, A. Cortegiani, F. Vitale, L. Pistidda, R. D‘Andrea, L. Querci, P. Gnesin, M. Todeschini, M. Lugano, G. Castelli, M. Ortolani, A. Cotoia, S. Maggiore, L. DiTizio, R. Graziani, I. Testa, E. Ferretti, C. Castioni, F. Lombardi, R. Caserta, M. Pasqua, S. Simoncini, F. Baccarini, M. Rispoli, F. Grossi, L. Cancelliere, M. Carnelli, F. Puccini, G. Biancofiore, A. Siniscalchi, C. Laici, E. Mossello, M. Torrini, G. Pasetti, S. Palmese, R. Oggioni, V. Mangani, S. Pini, M. Martelli, E. Rigo, F. Zuccalà, A. Cherri, R. Spina, I. Calamai, N. Petrucci, A. Caicedo, F. Ferri, P. Gritti, N. Brienza, R. Fonnesu, M. Dessena, G. Fullin, D. Saggioro

**Affiliations:** 1 Department Information Engineering, Università Degli Studi Di Brescia, via Branze 38, 25123 Brescia, Italy; 2 U.O. Medicina, ASST Spedali Civili, P.O. Montichiari, Montichiari, Italy; 3 Department of Rehabilitation and Aged Care, “Fondazione Camplani” Hospital, Cremona, Italy; 4 REFiT Bcn Research Group, Parc Sanitari Pere Virgili and Vall D’Hebron Institut de Recerca (VHIR), Barcelona, Spain; 5 Department of Systems Medicine, Tor Vergata University, Rome, Italy; 6 Department of Clinical and Behavioral Neurology, IRCCS Fondazione Santa Lucia, Rome, Italy; 7 Bicocca Center of Bioinformatics, Biostatistics and Bioimaging, Università Degli Studi Di Milano-Bicocca, Monza, Italy; 8 Geriatria, Accettazione Geriatrica e Centro Di Ricerca Per L’invecchiamento, POR, IRCCS INRCA, Ancona, Italy; 9 Section of Geriatrics, Department of Medical Sciences, Università Degli Studi Di Torino, A.O.U. Città Della Salute E Della Scienza Di Torino, Turin, Italy; 10 Department of Clinical and Experimental Medicine, Università Degli Studi Di Firenze, Firenze, Italy; 11 Department of Clinical and Experimental Sciences, Università Degli Studi Di Brescia, Brescia, Italy; 12 School of Medicine and Surgery, Università Degli Studi Milano-Bicocca and Acute Geriatric Unit, San Gerardo Hospital Monza, Milan, Italy

**Keywords:** Delirium, Older persons, Sarcopenia

## Abstract

**Introduction:**

Delirium and sarcopenia are common, although underdiagnosed, geriatric syndromes. Several pathological mechanisms can link delirium and low skeletal muscle mass, but few studies have investigated their association. We aimed to investigate (1) the association between delirium and low skeletal muscle mass and (2) the possible role of calf circumference mass in finding cases with delirium.

**Methods:**

The analyses were conducted employing the cross-sectional “Delirium Day” initiative, on patient 65 years and older admitted to acute hospital medical wards, emergency departments, rehabilitation wards, nursing homes and hospices in Italy in 2017. Delirium was diagnosed as a 4 + score at the 4-AT scale. Low skeletal muscle mass was operationally defined as calf circumference ≤ 34 cm in males and ≤ 33 cm in females. Logistic regression models were used to investigate the association between low skeletal muscle mass and delirium. The discriminative ability of calf circumference was evaluated using non-parametric ROC analyses.

**Results:**

A sample of 1675 patients was analyzed. In total, 73.6% of participants had low skeletal muscle mass and 24.1% exhibited delirium. Low skeletal muscle mass and delirium showed an independent association (OR: 1.50; 95% CI 1.09–2.08). In the subsample of patients without a diagnosis of dementia, the inclusion of calf circumference in a model based on age and sex significantly improved its discriminative accuracy [area under the curve (AUC) 0.69 vs 0.57, *p* < 0.001].

**Discussion and conclusion:**

Low muscle mass is independently associated with delirium. In patients without a previous diagnosis of dementia, calf circumference may help to better identify those who develop delirium.

**Supplementary Information:**

The online version contains supplementary material available at 10.1007/s40520-021-01950-8.

## Introduction

Delirium is a common and severe neuropsychiatric condition, characterized by an acute and fluctuating disorder of attention and cognitive function [1]. It is strongly associated with increased mortality, risk of re-hospitalization, institutionalization, higher costs of health services [1] and increased distress for patients, caregivers and health care providers [2, 3].

Older persons are at greater risk of developing delirium: between 25 and 33% of older adults who are admitted to a medical ward develop this syndrome, either as the presenting sign of underlying acute illnesses or during the hospitalization [4–6].

The reduction of muscle mass is another common condition among older persons: each decade after age 30, between 3 and 8% of muscle mass is involuntarily lost [7]. Low skeletal muscle mass (SMM) is often associated with low muscle strength and poor functional performance, configuring the diagnosis of sarcopenia [8]. Similarly to those with delirium, persons with sarcopenia have increased risk of death, hospitalization, and of developing disability and loss of autonomy [8]. Sarcopenia is a main feature of frailty [9], which has been found associated with a higher risk of delirium [10].

However, few studies [11] so far have evaluated the overlap and the association between delirium and low SMM. These two conditions might have common pathogenetic mechanisms, such as inflammation, nutritional deficit, and low mobility [12, 13]. Furthermore, they may influence each other when simultaneously present: for example, a reduction in muscle mass may alter the distribution volumes of drugs and thus their pharmacokinetic, enhancing the risk of drug-induced delirium [14]. Lastly, both conditions, although common and impactful, are often underdiagnosed [1, 8] even if rapid screening and diagnostic tools are available.

In this study, we aimed to investigate if low SMM, defined as a reduced calf circumference, is independently associated with delirium. Further, we aimed to investigate the possible role of calf circumference in finding cases with delirium.

## Materials and methods

### Study population

We analyzed data from the Italian Delirium Day, 2017 edition. The aim and the details of the Delirium Day studies have been described elsewhere [5, 6]. Briefly, the physicians belonging to 12 Italian scientific societies were invited to participate in the study. Data about patients older than 65 years and admitted to the participating centers were collected from 00:00 to 23:59 on the 27th of September 2017. Demographics and information about medical history and pharmacological treatment were retrieved through medical records review, interview, and physical examination. Length of stay was calculated as the number of days between admission and the index day. Patients affected by aphasia, blindness, deafness, in a coma or in terminal conditions were excluded. An informed consent form was signed by either the patient or, in case of delirium or severe cognitive decline, by a proxy. Detailed information about data collection methodologies was provided and all data were gathered by the participating physicians using a web-based form. The study was approved by the Brianza Ethics Committee (protocol no. 2572). This study complies with the Declaration of Helsinki ethical standards.

In total, 3751 patients were recruited for the 2017 Delirium Day edition. Those who did not met inclusion criteria (*n* = 341) were excluded. Further, 127 were excluded because they missed information about demographics or delirium and 1582 were excluded because of missing data on calf circumference. After excluding 26 patients because the recorded value of calf circumference was an outlier, a total sample of 1675 patients was analyzed (Figure S1).

### Delirium assessment

Delirium was assessed employing the 4AT [15], a rapid and validated tool for delirium detection. In a recent metanalysis, it showed high sensitivity and specificity (0.86 and 0.89, respectively) in the diagnosis of delirium against reference standards [16]. Shortly, the tool is based on 4 items investigating acute changes in alertness, attention, and cognition. The probability of delirium is stratified according to the final score of the 4AT as follows: absence of delirium (0 points), unlikely diagnosis (1–3 points) or probable diagnosis (4 + diagnosis). In our study, a 4AT score of 4 + points was used to diagnose delirium.

### Low skeletal muscle mass assessment

Calf circumference was measured employing a semi-rigid tape around the widest part of the calf, applying a minimum pressure in order not to compress the subcutaneous tissue.

Calf circumference was dichotomized to operationally define low SMM, using sex-specific cut-offs for its identification (≤ 34 cm for males, ≤ 33 cm for females) [17–19].

### Statistical analyses

Calf circumference outliers were identified by dividing the study population in 8 age-groups and assessing the distribution of calf circumference in each group: if the calf circumference was higher than the 3rd quartile or lower than the 1st quartile by more than three times the interquartile range, the value was considered an outlier and the participant was excluded.

Study population’s characteristics were described using absolute number and proportion, mean and standard deviation (SD) or median and interquartile range (IQR), as appropriate. Differences between patients with a diagnosis of delirium and those without were investigated by means of chi-squared test, t test or Mann–Whitney *U* test, as appropriate. The associations between calf circumference and delirium were evaluated employing logistic regression models, both unadjusted and adjusted for major confounders. To investigate the role of calf circumference in delirium detection, the discriminative ability of different logistic regression models has been assessed using the area under the curve (AUC) from non-parametric ROC analyses. The relative improvement in terms of goodness-of-fit between models was calculated as the difference between the AUC of the evaluated model and the one of the reference model, divided by the AUC of the reference model. The AUCs of the logistic regression models were compared using bootstrapping technique (*n* = 2000). Non-parametric ROC analyses were used to assess the AUC and sensitivity/specificity of calf circumference alone in the detection of delirium among participants without dementia, stratifying by sex.

All analyses were conducted with R 4.0.3 (R Foundation for Statistical Computing, Vienna, Austria [20]) with an alpha level = 0.05 (Table 1).

**Table 1:** Characteristics of the study population, stratified according to calf circumference

	Whole sample (*n* = 1675)	Without low skeletal muscle mass (*n* = 443) (26.4%)	With low skeletal muscle mass (*n* = 1232) (73.6%)	*p*
Age, mean (SD)	83.1 (7.6)	81.3 (7.6)	83.8 (7.5)	< 0.001
Male gender (%)	618 (36.9)	177 (40.0)	441 (35.8)	0.134
Setting (%)				0.075
Medical ward	789 (47.1)	195 (44.0)	594 (48.2)	
Nursing home	501 (29.9)	130 (29.3)	371 (30.1)	
Rehabilitation unit	272 (16.2)	86 (19.4)	186 (15.1)	
Surgical ward	82 (4.9)	27 (6.1)	55 (4.5)	
Palliative care unit	31 (1.9)	5 (1.1)	26 (2.1)	
Charlson’s comorbidity index, median (IQR)	3.0 (3.0)	2.0 (3.0)	3.0 (4.0)	< 0.001
Number of prescribed drugs, median (IQR)	7.0 (4.0)	7.0 (4.0)	7.0 (4.0)	0.361
Ischemic heart disease (%)	276 (16.5)	60 (13.5)	216 (17.5)	0.062
Heart failure (%)	412 (24.6)	106 (23.9)	306 (24.8)	0.751
Severe chronic kidney disease (%)	292 (17.4)	73 (16.5)	219 (17.8)	0.586
Dementia (%)	555 (34.8)	115 (27.6)	440 (37.4)	< 0.001
Length of stay, days, median (IQR)	12 (142.2)	12 (216.5)	12 (135.0)	0.880
Calf circumference, cm, median (IQR)	31.0 (6.0)	36.0 (3.0)	29.0 (4.0)	< 0.001
Delirium (%)	403 (24.1)	75 (16.9)	328 (29.3)	< 0.001

*SD* standard deviation, *IQR* interquartile range

## Results

The mean age of the 1675 participants was 83.1 and 36.9% were males. The study population was mostly recruited in medical wards (47.1%), followed by nursing homes (29.9%), rehabilitation units (16.2%), surgical wards (4.9%), and palliative care units (1.9%). The median calf circumference was 31.0 cm (IQR = 6.0 cm). The participants excluded from this study because of missing values of calf circumference were younger, more likely to be male, without a previous diagnosis of dementia, and to be admitted to a general surgery ward or an intensive care unit in comparison with those included (all *p* < 0.050). The prevalence of delirium was similar between the two groups (*p* = 0.514).

Calf circumference was associated with delirium, even after adjustment for age, sex, dementia diagnosis, CCI, recruitment setting, and ischemic heart disease diagnosis (Table 2). In the adjusted model, each centimeter more in calf circumference was associated with a 5% decrease in the probability of exhibiting delirium. Participants with low SMM were 1.5 times more likely to have delirium (adjusted odds ratio 95% confidence interval 1.50–1.09, 2.08).

**Table 2:** Associations of calf circumference measures with delirium

	Unadjusted OR (95%CI)	Adjusted OR (95%CI)
Calf circumference	0.95 (0.93–0.97)	0.95 (0.92–0.97)
Low skeletal muscle mass	1.78 (1.35–2.36)	1.50 (1.09–2.08)

Adjustment: age, sex, Charlson’s comorbidity index, dementia, ischemic heart disease, and setting

*OR* odds ratio, *95% CI* 95% Confidence intervals

A logistic regression model based on age, sex and dementia diagnosis showed a discriminative ability in identifying delirium cases of 0.69 (Table 3). Including in the model the Charlson’s comorbidity index, the length of stay (shown to be associated with delirium in univariate analyses, Table S1) and the calf circumference improved the goodness-of-fit by 15.9% (AUC 0.80, *p* < 0.001).

**Table 3:** goodness-of-fit (AUC) of different in logistic regression models in predicting delirium, in the whole sample and in the subsample of participants without a diagnosis of dementia

	Whole sample				Without a dementia diagnosis			
	OR (95%CI)	AUC	*p*	AUC relative improvement	OR (95%CI)	AUC	*p*	AUC relative improvement
Age	1.02 (1.00–1.04)	0. 69	Ref	Ref	1.03 (1.00–1.06)	0.57	Ref	Ref
Male Gender	1.19 (0.90–1.58)				1.33 (0.87–2.02)			
Dementia	9.06 (6.93–11.93)				–			
Age	1.02 (1.00–1.04)	0.78	< 0.001	13.0%	1.03 (1.00–1.06)	0.63	0.012	10.5%
Male Gender	1.14 (0.86–1.52)				1.19 (0.77–1.82)			
Dementia	9.14 (6.97–12.06)				–			
Charlson’s com. Index	1.04 (1.00–1.09)				1.08 (1.02–1.14)			
Length of stay	1.00 (1.00–1.00)				1.00 (1.00–1.00)			
Age	1.01 (1.00–1.03)	0.80	< 0.001	15.9%	1.03 (1.00–1.06)	0.69	< 0.001	21.0%
Male Gender	1.21 (0.91–1.62)				1.41 (0.90–2.20)			
Dementia	9.31 (7.08–12.34)				–			
Charlson’s com. Index	1.04 (0.99–1.08)				1.07 (1.01–1.13)			
Length of stay	1.00 (1.00–1.00)				1.00 (1.00–1.00)			
Calf circumference	0.94 (0.92–0.97)				0.90 (0.87–0.93)			

*OR* odds ratio, *95% CI* 95% Confidence Interval, *AUC* area under the curve

Among participants without a diagnosis of dementia, a logistic regression model based on age and sex showed a discriminative ability lower than 0.60. Including the Charlson’s comorbidity index and the length of stay lead to an AUC of 0.63. The further inclusion of the calf circumference improved the AUC of the model by more than 20%, in comparison with the reference one (AUC 0.69, *p* < 0.001).

**Fig. 1:**
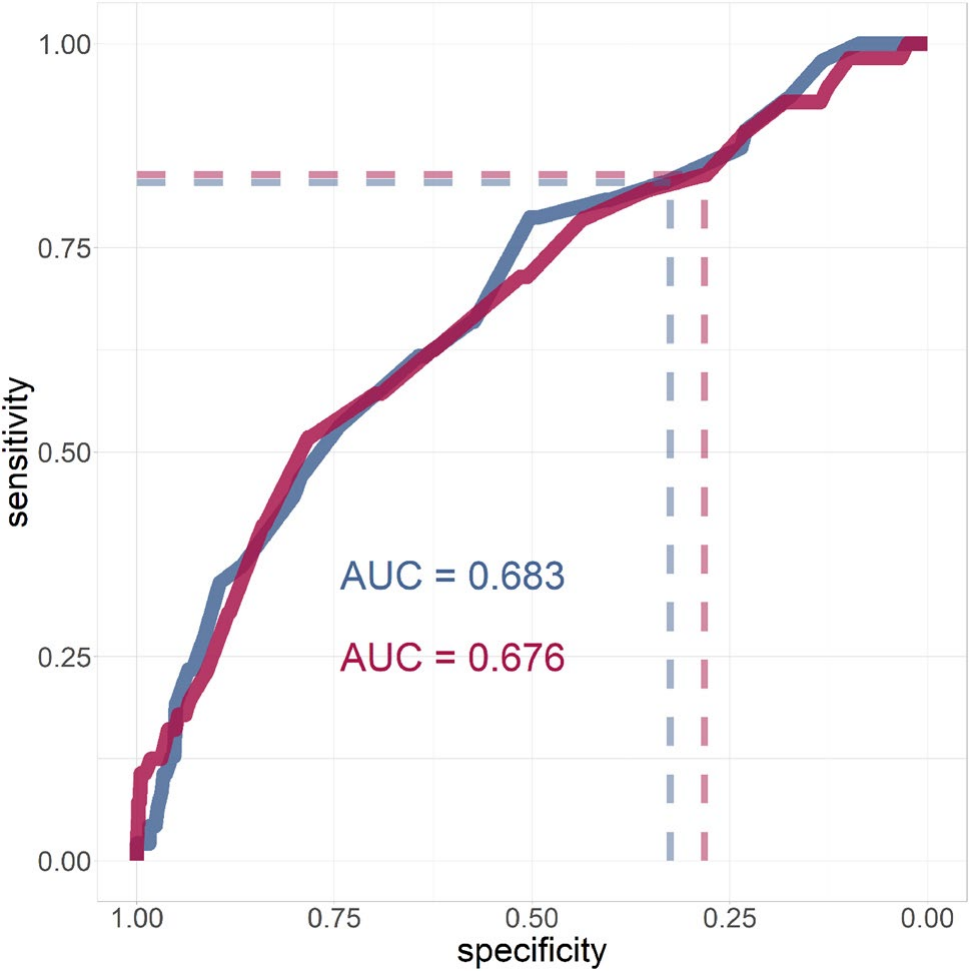
Receiver-Operating-Characteristic curve for the identification of delirium in the subsample of patients without a diagnosis of dementia, stratified by sex (blue = males, purple = females)

Figure 1 shows the ROC curves of calf circumference alone (unadjusted) in identifying delirium cases among patients without a diagnosis of dementia, in males and females. The AUC was 0.68 for both genders. The cut-offs used for the diagnosis of low SMM exhibited a sensitivity of 0.83 for both genders. Specificity was lower among females (0.28) than among males (0.32).

## Discussion

In this multi-center study, we show that sarcopenia, measured by means of calf circumference, is independently associated with delirium and that, in patients without a previous diagnosis of dementia, calf circumference may help to identify delirium cases.

In our study, the prevalence of delirium was similar to the one reported in the literature [20], confirming the association between delirium and age, dementia, and the presence of multiple chronic conditions as already described [21].

Conversely, the proportion of patients with low calf circumference was higher than the one previously reported (ranging between 7 and 24% [19, 22–26]). In most of these studies, the mean calf circumference exhibited by those affected by low skeletal muscle mass (range 31.5–32.4 cm) was higher than the one we found. In one study [25], including black South African women, the mean calf circumference for those diagnosed with low SMM according to DXA (28 cm) was similar the one found in our sample. Most studies, however, enrolled participants living in the community. Indeed, this finding may be, at least partially, explained by the fact that our study population was on average older than 80 years old, admitted to a hospital ward or living in nursing homes, and with high prevalence of chronic conditions and dementia, given the association of all these factors with low muscle mass and sarcopenia [8, 27–29].

A previous study [11] reported an association between sarcopenia (defined according to EGWSOP criteria) and delirium in hospitalized patients in Italy, confirming our results. Several pathogenic mechanisms might explain the association we found between low SMM and delirium. Low muscle mass and sarcopenia are strongly linked with frailty, in particular with its physical phenotype [9, 30]: patients with lower calf circumference might be affected by frailty and, as such, at higher risk of developing delirium [10]. Furthermore, inflammatory cytokines and markers have been shown in the serum of persons with delirium [31] and in those affected by sarcopenia and low muscle mass [32]: abnormal and dysregulated inflammatory response might serve as trigger to both conditions. Changes in body composition typical of sarcopenic patients have been associated with increased risk of adverse drug reactions [14]: in consideration of the strong association between polypharmacy and delirium [33], it is likely that patients with reduced muscle mass are at higher risk of developing delirium even when therapeutic drug dosage are used. Our study strengthens previous results about the association of low muscle mass and delirium, broadening these findings to calf circumference, a simple proxy proposed for the evaluation of muscle quantity in the diagnostic algorithm of sarcopenia.

Of notice, among participants without a known diagnosis of dementia (26.8% of delirium cases), calf circumference significantly improved the AUC of a model based on age, sex, comorbidities, and length of staying. Further, calf circumference alone showed a moderate discriminative ability in identifying delirium cases among participants without dementia. It is likely that when an overt dementia diagnosis is present, the discriminative ability of calf circumference in identifying those with delirium is weakened because of the strong association between dementia and delirium, which may overshadow the association between low SMM and delirium. Conversely, it is possible that, among those without a known diagnosis of dementia, the discriminative ability of calf circumference for delirium identification is higher, not only because of the aforementioned mechanisms, but also because it may identify those with prodromal or undiagnosed dementia, as lower muscle mass, sarcopenia, and cognitive impairment are reciprocally associated [24].

The results of our study should be read in light of some limitations. First, the Delirium Day is a point-prevalence study, preventing the possibility to investigate the causal relationship between low SMM and delirium. Second, although a high correlation between calf circumference and SMM has been reported [19, 22, 34], participants with oedema, obesity, or other conditions characterized by increased calf circumference and decreased muscle mass could not be correctly identified, thus possibly hampering the reliability of such measure in a minority of patient [34, 35].

Some study strengths may also be considered. This is the first multi-center study identifying such association in a large cohort of older patients from several settings of care. Moreover, the diagnosis of delirium was obtained with a well-validated screening tool, which does not require preliminary training to be administered.

Our study has implications for the everyday clinical practice. Calf circumference is simple to measure by all healthcare workers in hospital and long-term care wards. For example, the calf circumference cut-offs suggested for the identification of low SMM exhibited high sensitivity in the identification of delirium cases. If future studies will confirm the study findings, calf circumference could be proposed as a method to identify patients at risk of developing delirium and other adverse outcomes.

## Conclusion

Calf circumference is a rapid, easy to perform, and economic tool for SMM quantity evaluation: the inclusion of such simple measure in clinical practice might help to find cases of delirium, in particular those without manifest risk factors for delirium, such as dementia.

## Supplementary Information

Below is the link to the electronic supplementary material.

Supplementary file1 (DOCX 14 kb)

Supplementary file2 (DOCX 24 kb)

Supplementary file3 (TIF 2436 kb)

## Declarations

### Transparency statement

The lead authors (AZ and FM) affirm that the manuscript is an honest, accurate, and transparent account of the study being reported; that no important aspects of the study have been omitted; and that any discrepancies from the study as originally planned have been explained.
