# Supplementary material for: The association between low skeletal muscle mass and delirium: results from the nationwide multi-centre Italian Delirium Day 2017
Source: Aging Clin Exp Res. 2021 Aug 20;34(2):349–57. doi: 10.1007/s40520-021-01950-8 (PMC8847195; doi:10.1007/s40520-021-01950-8)
Supplement: Supplementary file 1 — Supplementary file1 (DOCX 14 kb) [file 40520_2021_1950_MOESM1_ESM.docx]

Table S1: study population characteristics in the whole sample and stratified by delirium diagnosis

|  | *Without delirium*  *N = 1272 (75.9%)* | *With delirium*  *N = 403 (24.1%)* | *p* |
| --- | --- | --- | --- |
| Age, mean (SD) | 82.45 (7.80) | 85.22 (6.67) | <0.001 |
| Male gender (%) | 483 (38.0) | 135 (33.5) | 0.118 |
| Setting (%) |  |  | <0.001 |
| *Medical ward* | 611 (48.0) | 178 (44.2) |  |
| *Nursing home* | 344 (27.0) | 157 (39.0) |  |
| *Rehabilitation unit* | 233 (18.3) | 39 (9.7) |  |
| *Surgical ward* | 64 (5.0) | 18 (4.5) |  |
| *Palliative care unit* | 20 (1.6) | 11 (2.7) |  |
| Charlson’s comorbidity index, median (IQR) | 3.0 (3.0) | 3.0 (2.5) | 0.010 |
| Number of prescribed drugs, median (IQR) | 7.0 (4.0) | 7.0 (4.0) | 0.546 |
| Ischemic heart disease (%) | 215 (16.9) | 61 (15.1) | 0.450 |
| Heart failure (%) | 320 (25.2) | 92 (22.8) | 0.379 |
| Severe chronic kidney disease (%) | 221 (17.4) | 71 (17.6) | 0.970 |
| Dementia (%) | 273 (22.6) | 282 (73.2) | <0.001 |
| Length of stay, days, median (IQR) | 11 (52.0) | 16 (504.25) | <0.001 |
| Calf circumference, cm, median (IQR) | 31.0 (7.0) | 30.0 (6.25) | <0.001 |
| Low SMM (%) | 904 (71.1) | 328 (81.4) | <0.001 |

Abbreviations: SD = standard deviation; IQR = interquartile range, SMM: skeletal muscle mass
