# Supplementary material for: The association between low skeletal muscle mass and delirium: results from the nationwide multi-centre Italian Delirium Day 2017
Source: Aging Clin Exp Res. 2021 Aug 20;34(2):349–57. doi: 10.1007/s40520-021-01950-8 (PMC8847195; doi:10.1007/s40520-021-01950-8)
Supplement: Supplementary file 2 — Supplementary file2 (DOCX 24 kb) [file 40520_2021_1950_MOESM2_ESM.docx]

**Supplementary Material 2.**

*Steering Committee:* Giuseppe Bellelli (Chair, University of Milano-Bicocca, Milan), Alessandro Morandi (Department of Rehabilitation and Aged Care, “Fondazione Camplani” Hospital, Cremona, Italy), Alessandra Marengoni (Department of Clinical and Experimental Sciences, University of Brescia, Brescia, Italy), Enrico Mossello (Research Unit of Medicine of Ageing, Department of Experimental and Clinical Medicine, University of Florence and Azienda Ospedaliero-Universitaria Careggi, Florence, Italy), Antonio Cherubini (Geriatria, Accettazione geriatrica, Centro di ricerca per l’invecchiamento, IRCCS-INRCA, Ancona, Italy).

*Clinical data monitoring and revision:* Simona Di Santo (Department of Clinical and Behavioral Neurology, Neuropsychiatry Laboratory, IRCCS Foundation S Lucia, Rome, Italy), Elena Tassistro, Emanuela Rossi, Anita Andreano, Maria Grazia Valsecchi (Bicocca Center of Bioinformatics, Biostatistics and Bioimaging (B4 center), University of Milano-Bicocca, Monza, Italy).

*Database Management and Statistics:* Elena Tassistro, Anita Andreano, Maria Grazia Valsecchi (Bicocca Center of Bioinformatics, Biostatistics and Bioimaging (B4 center), University of Milano-Bicocca, Monza, Italy).

*Investigators:*

Tarasconi A, Unità Operativa Chirurgia d'Urgenza, Azienda Ospedaliero-Universitaria Di Parma; Sella M, Auriemma S, Paternò G, Aulss 8 Berica, Vicenza; Faggian G, Lucarelli C, Azienda Ospedaliero-Universitaria di Verona; De Grazia N, Alberto C, Chirurgia AO Pugliese Ciaccio Di Catanzaro; Margola A, 1^ Chirurgia Asst Spedali Civili di Brescia; Porcella L, Nardiello I, 3^ Chirurgia Asst Spedali Civili di Brescia; Chimenti E, Zeni M, 2^ Chirurgia Spedali Civili di Brescia; Giani A, Famularo S, Ospedale San Gerardo; Romairone E, Minaglia C, IRCCS Ospedale Policlinico San Martino, Genova; Ceccotti C, Guerra G, Mantovani G, Monacelli F, Minaglia C, Clinica Geriatrica Ospedale Policlinico San Martino, Genova; Candiani T, Medicina Generale - Geriatria; Ballestrero A, Minaglia C, Ospedale Policlinico San Martino, Genova; Santolini F, Minaglia C, Ospedale Policlinico San Martino, Genova; Rosso M, Bono V, Neurologia Ospedale Ss Annunziata; Sibilla S, Azienda Ospedaliera Pia Fondazione "Card. G. Panico"; Dal Santo P, Ceci M, Unità Operativa Complessa Geriatria Ulss 5 "Polesana"; Barone P, Schirinzi T, Azienda Ospedaliera Pia Fondazione "Card. G.Panico"; Formenti A, Nastasi G, Azienda Ospedaliera San Gerardo, Monza; Isaia G, Geriatria Azienda Ospedaliero-Universitaria San Luigi Gonzaga di Orbassano; Gonella D, Ospedale Galliera, Genova; Battuello A, Casson S, Ospedale di Chioggia Ulss 3 Serenissima, Mestre; Calvani D, Boni F, Soc Geriatria Nuovo Ospedale Santo Stefano - Prato- Azienda Asl Toscana Centro; Ciaccio A, Rosa R, Gastroenterologia; Sanna G, Manfredini S, Ospedale Civile Ss. Annunziata; Cortese L, Campus Bio-Medico di Roma; Rizzo M, Prestano R, Vi Medicina Interna Università della Campania Luigi Vanvitelli; Greco A, Lauriola M, S.C. di Geriatria; Gelosa G, Piras V, Grande Ospedale Metropolitano Niguarda; Arena M, Cosenza D, UOSD Stroke Unit; Bellomo A, LaMontagna M, S.P.D.C. Foggia, Asl Foggia, Ospedale Riuniti Foggia; Gabbani L, Lambertucci L, Sod Geriatria per La Complessità Assistenziale - Azienda Ospedaliero-Universitaria Careggi; Perego S, Parati G, Istituto Auxologico Italiano Ospedale San Luca; Basile G, Uosd Geriatria; Gallina V, Pilone G, Azienda Ospedaliera Villa Scassi- Asl3; Giudice C, De F, Geriatria; Pietrogrande L, De B, Asst Santi Paolo E Carlo, Presidio S. Paolo; Mosca M, Corazzin I, Uo Complessa di Medicina Ospedale di Agordo; Rossi P, Nunziata V, Fondazione Irccs Ca' Granda Ospedale Maggiore Policlinico; D'Amico F, Grippa A, Unità Operativa Complessa Geriatria Po "Barone Ignazio Romeo " Patti; Giardini S, Reparto Geriatria per Acuti Ospedale Santa Maria Annunziata Usl Toscana Centro; Barucci R, Ortogeriatria Ospedale Santa Maria Annunziata Usl Toscana Centro; Cossu A, Fiorin L, Unità Operativa Medicina, Ospedale G.P. Delogu; Arena M, Distefano M, Uosd Neurofisiopatologia E Disordini del Movimento; Lunardelli M, Brunori M, Uo Geriatria; Ruffini I, Abraham E, Reparto di Geriatria Merano; Varutti A, Fabbro E, Degenze Chirurgiche; Catalano A, Martino G, Policlinico Universitario "G. Martino" di Messina; Leotta D, Marchet A, Struttura Complessa Neurologia; Dell'Aquila G, Scrimieri A, Inrca; Davoli M, Casella M, Arcispedale Santa Maria Nuova -Irccs - Soc Geriatria; Cartei A, Polidori G, Medicina Interna E Post-Chirurgica - Azienda Ospedaliero Universitaria Careggi; Basile G, Brischetto D, Unità Operativa Complessa Medicina Delle Malattie Metaboliche; Motta S, U.O Cardiologia - Asst Monza; Saponara R, Asst Crema, Ospedale Maggiore; Perrone P, Asst Ovest Milanese; Russo G, A.O.U Federico Ii Di Napoli; Del D, Car C, Unità Operativa Complessa Neurologia, Ospedale Versilia, Azienda Usl Toscana Nord Ovest; Pirina T Unità Operativa di Geriatria; Franzoni S, Dipartimento Ortopedia E Traumatologia Fondazione Poliambulanza Istituto Ospedaliero; Cotroneo A, Ghiggia F, Reparto Geriatria Obdv; Volpi G, Menichetti C, Ospedale San Iacopo Pistoia; Bo M, Panico A, A.O.U. Città della Salute e della Scienza, Torino; Calogero P, Corvalli G, Geriatria Calogero Policlinico Sant'Orsola Malpighi; Mauri M, Ospedale Circolo e Fondazione Macchi Varese; Lupia E, Medicina D'Urgenza - Mecau - A.O.U. Città della Salute e della Scienza, Torino; Manfredini R, Fabbian F, Clinica Medica; March A, Pedrotti M, Geriatria Ospedale Di Bolzano; Veronesi M, Strocchi E, UO Medicina Interna; Bianchetti A, Crucitti A, Istituto Clinico S. Anna, Brescia; Di Francesco V, Fontana G, Geriatria A Azienda Ospedaliera Universitaria Integrata Verona; Bonanni L, Barbone F, Clinica Neurologica Università di Chieti; Serrati C, UO Neurologia Ospedaliera con Centro Ictus, Savona; Ballardini G, Simoncelli M, Medicina Rimini; Ceschia G, Scarpa C, Struttura Complessa Geriatria; Brugiolo R, Fusco S, Unità Operativa Complessa Geriatria - Ospedale Dell'Angelo, Trieste; Ciarambino T, Medicina Interna Marcianise; Biagini C, Tonon E, Unità Operativa Complessa Geriatria; Porta M, Sc Medicina 1U - Molinette Torino; Venuti D, Ospedale Evangelico Internazionale Genova; DelSette M, Poeta M, Ente Ospedaliero Ospedali Galliera, Genova; Barbagallo G, Trovato G, Medicina Interna P.O. Basilotta Nicosia (En) Asp 4, Enna; Delitala A, Clinica Medica - Azienda Ospedaliero-Universitaria di Sassari; Arosio P, Reggiani F, Humanitas Research Hospital, Rozzano, Milano; Zuliani G, Ortolani B, Arcispedale Sant'Anna di Cona; Mussio E, Fondazione Poliambulanza Istituto Ospedaliero, Brescia; Girardi A, Coin A, Clinica Geriatrica, Padova; Ruotolo G, Castagna A, Geriatria A.O. Pugliese Ciaccio di Catanzaro; Masina M, Unità Operativa Complessa Geriatria Bentivoglio; Cimino R, Soc Medicina Interna; Pinciaroli A, Soc Nefrologia Ao Pugliese Ciaccio di Catanzaro; Tripodi G, Soc Ortopedia; Cannistrà U, Soc Neurologia; Cassadonte F, Vatrano M, Utic Ao Pugliese Ciaccio di Catanzaro; Cassandonte F, Soc Cardiologia; Scaglione L, Medicina Interna 5; Fogliacco P, Muzzuilini C, Medicina Generale Presidio Ospedaliero Di Ceva Aslcn1; Romano F, S.C. Medicina Interna; Padovani A, Rozzini L, UO Neurologia Asst Spedali Civili di Brescia; Cagnin A, Fragiacomo F, Clinica Neurologica, Dipartimento di Neuroscienze, Università Di Padova; Desideri G, Liberatore E, Ospedale Ss. Filippo E Nicola; Bruni A, Orsitto G, UOC. Medicina Interna P.O. "di Venere", L’Aquila; Franco M, Bonfrate L, Unità Operativa Complessa Geriatria Ospedale Generale Regionale F. Miulli; Bonetto M, S.C. Geriatria; Pizio N, Uo Neurologia; Magnani G, Cecchetti G, Ospedale San Raffale, Milano; Longo A, Bubba V, S.C. Geriatria; Marinan L, Ospedale San Bassiano; Cotelli M, Turla M, Asst Valcamonica; Brunori M, Medicina Interna Zoli; Sessa M, Abruzzi L, UO Neurologia Asst Cremona Ospedale Di Cremona; Castoldi G, LoVetere D, Unità Operativa Complessa Ortopedia e Traumatologia, Vimercate; Musacchio C, Ortogeriatria Eo Galliera; Novello M, Cavarape A, Clinica Medica; Bini A, Leonardi A, Asl Roma 1, Presidio Ospedaliero S. Filippo Neri Unità Operativa Complessa Medicina Interna, Roma; Seneci F, 1°Traumatologia-Ortopedia Asst Spedali Civili di Brescia; Grimaldi W, Unità Operativa Geriatria Ospedale di Magenta (Mi) Asst Ovest Milanese; Seneci F, 2°Traumatologia-Ortopedia Asst Spedali Civili di Brescia; Fimognari F, Bambara V, UOC. Geriatria Azienda Ospedaliera di Cosenza; Saitta A, Corica F, Medicina Interna; Braga M, Neurologia Asst Vimercate; Servi Medicina Interna Universitaria; Ettorre E, Camellini C, Policlinico Umberto I; Marengoni A, Spedali Civili Presidio Montichiari, Brescia; Bruni A, Crescenzo A, Ospedale Giovanni Paolo Ii Asp Cz; Noro G, Turco R, Ospedale Santa Chiara, Trento; Ponzetto M, Giuseppe L, Ospedale Civico di Chivasso Asl To4, Torino; Mazzei B, Maiuri G, Irccs_Inrca Ospedale Cosenza Unità Operativa Complessa di Geriatria; Costaggiu D, Medicina Generale; Damato R, Fabbro E, Ass3 “Alto Friuli-Collinare-Mediofriuli” – Presidio Ospedaliero Sd-T Degenze Internistiche Sd; Formilan M, Az. Ulss 3 Geriatria Dolo; Patrizia G, Santuari L, Fondazione Sanità e Ricerca; Gallucci M, Minaglia C, Ospedale Policlinico San Martino, Genova; Paragona M, Fondazione Hospice M.T.C. Seràgnoli Onlus; Bini P, Fondazione Terasa Camplani Cdc Ancelle; Modica D, Hospice Kairòs Siracusa; Abati C, Clerici M, Casa Hospice Cima Verde; Barbera I, Hospice Via Delle Stelle; NigroImperiale F, Hospice San Giuseppe Moscati; Manni A, Votino C, "Hospice Casa Madonna Dell'Uliveto" Scsrl; Castiglioni C, Di M, Asst Ovest Milanese; Degl'Innocenti M, Moscatelli G, Hospice Di Abbiategrasso; Guerini S, Fondazione Ips Card. Gusmini Onlus; Casini C, Hospice Convento Delle Oblate- Ufc Cure Palliative-Usl Toscana Centro-Firenze; Dini D, Unità Operativa di Terapie Palliative/Hospice; D'Imporzano E, Hospice Livorno; DeNotariis S, Pronto Soccorso E Medicina D'Urgenza, Policlinico Sant'Orsola Malpighi; Bonometti F, Pronto Soccorso Gardone Val Trompia, Brescia; Paolillo C, Riccardi A, Azienda Sanitaria Universitaria Integrata di Udine; Tiozzo A, SamySalamaFahmy A, Ospedale di Chioggia Ulss 3 Serenissima; Riccardi A, Paolillo C, Reparto di Medicina D'Urgenza; DiBari M, Vanni S, Pronto Soccorso Ospedale Careggi, Firenze; Scarpa A, Zara D, Ospedale Villa Salus; Ranieri P, Istituto Clinico S. Anna; Calogero P, Corvalli G, Pare (Post Acuti Riabilitazione Estensiva) Policlinico Sant'Orsola Malpighi; Pezzoni D, Fondazione Nobile Paolo Richiedei, Gussago Brescia; Gentile S, Morandi A, Platto C, D'Ambrosio V, Faraci B, Brambilla C. Fondazione Teresa Camplani - Casa di Cura Ancelle della Carità - Cremona; Ivaldi C, Cure Intermedie Ospedale Gallino; Milia P, DeSalvo F, Istitut Prosperius Tiberino; Solaro C, Strazzacappa M, Casa Di Cura "Mons. Luigi Novarese"; Bo M, Panico A, Istituto Di Riposo per La Vecchiaia I.R.V; Cazzadori M, Confente S, Lungodegenza San Bonifacio, Verona; Bonetto M, S.S. Cure Intermedie; "Grasso M, Troisi E, IRCCS Fondazione Santa Lucia; " Magnani G, Cecchetti G, Ospedale San Raffaele, Milano; Guerini V, Ips Fondazione Cardinal Gusmini di Vertova; Bernardini B, Corsini C, Neuroriabilitazione Humanitas, Rozzano, Milano; Boffelli S, Fondazione Poliambulanza, Brescia; Filippi A, Delpin K, Lungodegenza Post Acuzie; Bertoletti E, Ospedale Santa Viola; Vannucci M, Tesi F, Casa Di Cura Villa Ulivella E Glicini; Crippa P, Malighetti A, Fondazione Camplani Domus Salutis, Brescia; "Caltagirone C, DiSant S, IRCCS Fondazione Santa Lucia, Roma; " Bettini D, Maltese F, Spedali Civili Presidio Montichiari – Subacuti, Brescia; Formilan M, Az. Ulss 3 Lungodegenza Geriatrica Dolo; Abruzzese G, Minaglia C, Irccs Ospedale Policlinico San Martino; Cosimo D, Azienda Ospedaliera Pia Fondazione "Cardinale G.Panico"; Azzini M, Cazzadori M, Azienda Ulss 9 Scaligera, Verona; Colombo M, Procino G, Istituto Geriatrico "Camillo Golgi"; Fascendini S, Barocco F, Centro di Eccellenza Alzheimer - Ferb Onlus - Ospedale Di Gazzaniga (Bergamo); Del P, Azienda Ospedaliera Pia Fondazione "Card. G.Panico"; D'Amico F, Grippa A, Uo Lungodegenza Po "Barone Ignazio Romeo" Patti; Mazzone A, Riva E, Istituto Piero Redaelli, Milano; Dell'Acqua D, Cottino M, Istituto Golgi Abbiategrasso, Pavia; Vezzadini G, Avanzi S, Ics Maugeri Castelgoffredo; Orini S, IRCCS Fatebenefratelli S. Giovanni Di Dio, Brescia; Sgrilli F, Mello A, Presidio P.Thouar; Lombardi L, Fondazione Istituto Ospedaliero di Sospiro Onlus, Cremona; Muti E, Fondazione Mons. A. Mazzali Onlus Mantova; Dijk B, Rsa Chiavari; Fenu S, Pes C, Policlinico Sassarese; Gareri P, Castagna A, CDCD Catanzaro Lido; Passamonte M, De F, Cure Sub Acute; Rigo R, Locusta L, Villaggio Amico, Gerenzano Varese; Caser L, Rosso G, Casa Della Divina Provvidenza Cottolengo di Biella; Cesarini S, Fontenuovo - Residenze Di Ospitalità Per Anziani - Fondazione Onlus; Cozzi R, Santini C, Rsa Virgilio Ferrari; Carbone P, Cazzaniga I, Rsa Oasi Domenica; Lovati R, Cantoni A, Rsa "Casa Per Coniugi"; Ranzani P, Rsa G.Gemellaro Onlus; Barra D, Pompilio G, Apsp Opera Romani; Dimori S, Fondazione Angelo Poretti E Angelo Magnani; Cernesi S, Riccò C, Cra Casa Serena; Piazzolla F, Capittini E, Rsa Parco Del Welfare; Rota C, Gottardi F, I.P.S. Fondazione Cardinal Gusmini; Merla L, Fondazione Ips Cardinal Gusmini Onlus, Vertova, Bergamo; Barelli A, Rsa Monticello; Millul A, Istituti Riuniti Airoldi E Muzzi; De G, Morrone G, C.R.A. "E. Cialdini"; Bigolari M, Minaglia C, Ospedale Policlinico San Martino; Macchi M, Zambon F, Piccola Casa Della Divina Provvidenza (Cottolengo); D'Amico F, Rsa Sant'Angelo Di Brolo; D'Amico F, Rsa "San Giovanni Di Dio" - Patti; Pizzorni C, Rsa Celesia Mantenimento; DiCasaleto G, Residenza San Gottardo; Menculini G, Marcacci M, Residenza Protetta "Creusa Brizi Bittoni"; Catanese G, Sprini D, Rsa Buon Pastore; DiCasalet T, Casa Di Riposo San Gottardo, Genova; Bocci M, Centro Di Rianimazione; Borga S, Caironi P, Scdu Anestesia E Rianimazione; Cat C, Cingolani E, Rianimazione 1; Avalli L, Greco G, Terapia Intensiva Cardio-Toraco-Vascolare; Citerio G, Gandini L, Asst Monza Ospedale San Gerardo, Monza; Cornara G, Lerda R, Anestesia E Rianimazione; Brazzi L, Azienda Ospedaliero-Universitaria Città della Salute e della Scienza di Torino; Simeone F, Caciorgna M, Terapia Intensiva Cardiotoracica Azienda Ospedaliera Senese; Alampi D, Azienda Ospedaliera Sant'Andrea; Francesconi S, Beck E, ASST Monza - Ospedale di Desio - Terapia Intensiva Generale; Antonini B, Vettoretto K, Ospedale Di Manerbio ASST del Garda; Meggiolaro M, Azienda Ospedaliera Universitaria Padova - Rianimazione Centrale; Garofalo E, Bruni A, Azienda Ospedaliero-Universitaria Mater Domini; Notaro S, Terapia Intensiva Cardio Respiratoria-Ticre-Ospedale Monaldi; Varutti R, Bassi F, Aas5 Friuli Occidentale; Mistraletti G, Marino A, Ospedale San Paolo; Rona R, Rondelli E, Rianimazione Generale Ospedale San Gerardo, Monza; Riva I, Anestesia 3 Ti Adulti; Scapigliati A, Policlinico Gemelli; Cortegiani A, Vitale F, Policlinico Paolo Giaccone. Università degli Studi di Palermo; Pistidda L, Cliniche Universitarie Azienda Ospedaliero-Universitaria - Sassari; D'Andrea R, Querci L, Policlinico Universitario Sant'Orsola-Malpighi; Gnesin P, Todeschini M, Terapia Intensiva - ASST Franciacorta, Brescia; Lugano M, Clinica Di Anestesia E Rianimazione Azienda Sanitaria Universitaria Integrata Udine; Castelli G, Ortolani M, Asst Mantova Ospedale Carlo Poma, Mantova; Cotoia A, Azienda Universitario Ospedaliera O.O.R.R.; Maggiore S, DiTizio L, Ospedale Ss. Annunziata; Graziani R, Testa I, unità Operativa Complessa Anestesia Rianimazione Ospedale Profili Di Fabriano (Ancona)-Asur Marche Av2; Ferretti E, Castioni C, Ospedale San Giovanni Bosco; Lombardi F, Caserta R, Fondazione Poliambulanza, Brescia; Pasqua M, Simoncini S, Asst Vallecamonica; Baccarini F, Unità Operativa Complessa Anestesia e Rianimazione - Ravenna; Rispoli M, Azienda Ospedaliera Dei Colli - Ospedale Monaldi, Napoli; "Grossi F, Cancelliere L, Centro di Rianimazione Scdu Anestesia E Rianimazione Azienda Ospedaliero-Universitaria Maggiore Della Carità - Novara; " Carnelli M, Asst Lariana; Puccini F, Biancofiore G, Anestesia E Rianimazione Trapianti- Azienda Ospedaliero Universitaria Pisana; Siniscalchi A, Laici C, Uo Anestesia e Terapia Intensiva Polivalente e Dei Trapianti - Faenza; Mossello E, Torrini M, Geriatria-UTIC Careggi, Firenze; Pasetti G, San Giovanni Di Dio; Palmese S, Azienda Ospedaliera Universitaria San Giovanni di Dio e Ruggi D'Aragona; Oggioni R, Mangani V, Terapia Intensiva Ospedale S. Giovanni di Dio Firenze; Pini S, Martelli M, Azienda Ospedaliera Universitaria Pisana. Rianimazione Pisa; Rigo E, Terapia Intensiva Neurochirugica Treviso; Zuccalà F, Cherri A, 1ª Unità di Terapia Intensiva Asst-Spedali Civili Di Brescia; Spina R, Calamai I, Anestesia E Rianimazione Presidio di Empoli; Petrucci N, Caicedo A, Terapia Intensiva, Asst-Garda, Brescia; Ferri F, Gritti P, - Terapia Intensiva Neurochirurgica - Asst Papa Giovanni XXIII, Bergamo; Brienza N, Terapia Intensiva "De Blasi" Azienda Ospedaliera Universitaria Policlinico Bari; Fonnesu R, Dessena M, Ospedale Giovanni Paolo II; Fullin G, Saggioro D, Ospedale Dell'Angelo - Mestre;
