# Supplementary figures and images for: The association between low skeletal muscle mass and delirium: results from the nationwide multi-centre Italian Delirium Day 2017
Source: Aging Clin Exp Res. 2021 Aug 20;34(2):349–57. doi: 10.1007/s40520-021-01950-8 (PMC8847195; doi:10.1007/s40520-021-01950-8)

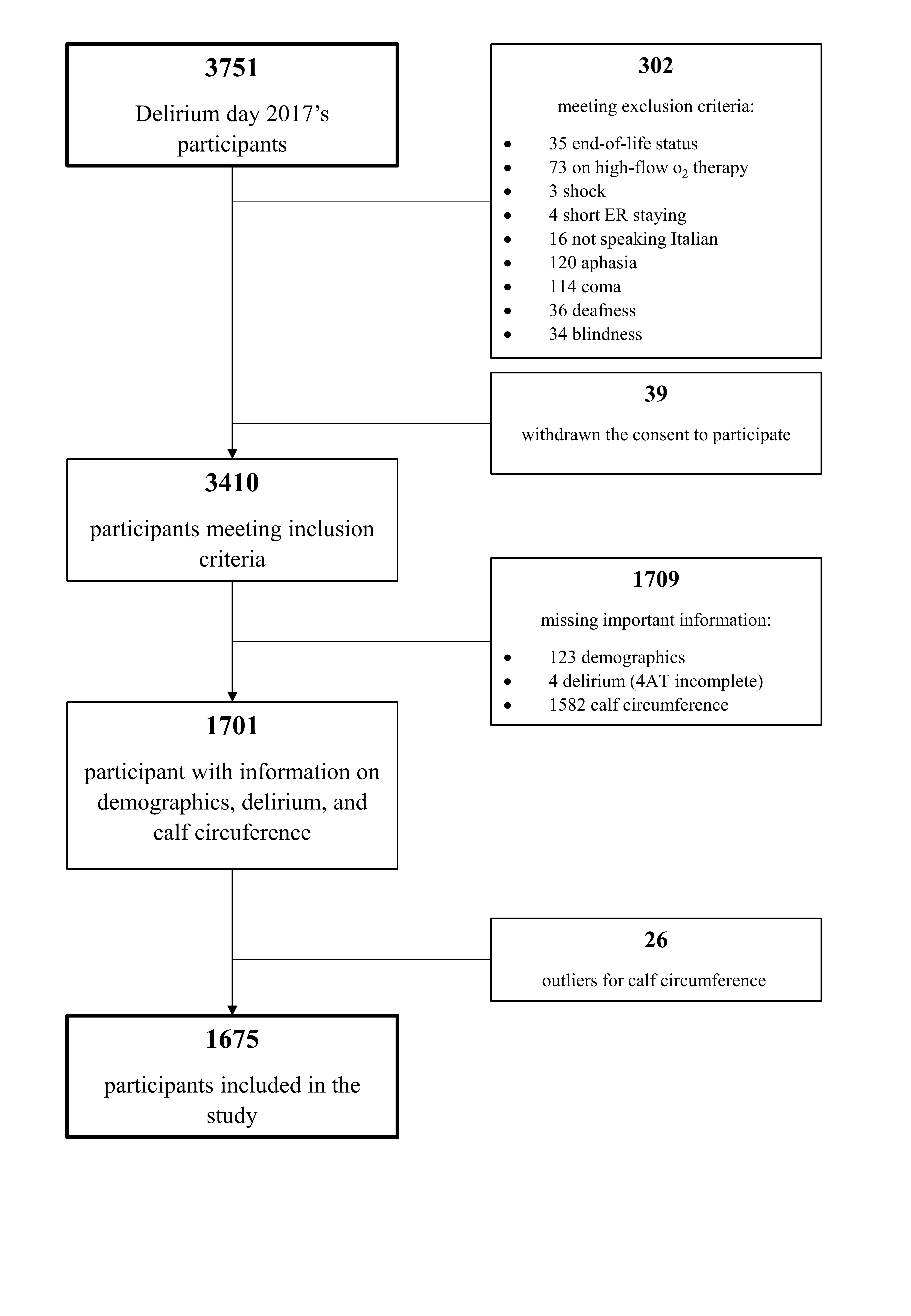

Supplement: Supplementary file 3 — Supplementary file3 (TIF 2436 kb) [file 40520_2021_1950_MOESM3_ESM.tif]
